# The ins and outs of breathing

**DOI:** 10.7554/eLife.03375

**Published:** 2014-06-17

**Authors:** Jan-Marino Ramirez, Tatiana M Anderson, Alfredo J Garcia

**Affiliations:** 1 **Jan-Marino Ramirez** is at the Center for Integrative Brain Research, Seattle Children's Hospital and Departments of Neurological Surgery and Pediatrics, University of Washington, Seattle, United Statesnino1@uw.edu; 2 **Tatiana M Anderson** is at the Center for Integrative Brain Research, Seattle Children's Hospital and Neurobiology and Behavior Graduate Program, University of Washington, Seattle, United States; 3 **Alfredo J Garcia III** is at the Center for Integrative Brain Research, Seattle Children's Research Institute, Seattle, United States

**Keywords:** breathing, Central pattern Generator, PreBötzinger complex, oscillator, Transcription, Mouse

## Abstract

Distinct populations of neurons within the brainstem are responsible for generating and coordinating the rhythmic patterns of neural activity that underlie breathing.

**Related research article** Tupal S, Huang W-H, Picardo MCD, Ling G-Y, Del Negro CA, Zoghbi HY, Gray PA. 2014. *Atoh1*-dependent rhombic lip neurons are required for temporal delay between independent respiratory oscillators in embryonic mice. *eLife* **3**:e02265. doi: 10.7554/eLife.02265

**Image** The rhythms of nerve impulses to different muscles need to be coordinated for breathing

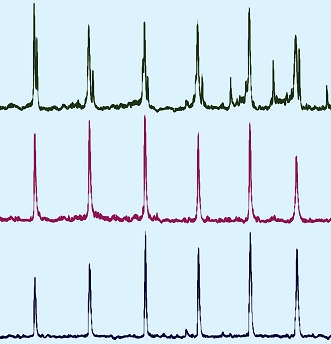

From our first breath to our last we continuously maintain and adjust our breathing to meet our metabolic, behavioural and environmental needs. For example, we breathe faster when we are anxious or while we are working out; we gasp when we run out of air, and we sigh when we are relieved. Yet, despite all of its complexity, breathing is controlled by a very small network of neurons within the brainstem (Richter and Smith, 2014).

Our breathing follows a rhythm that can have up to three phases. The first phase, which is called ‘inspiration’, begins when we breathe in: our diaphragm and some of the muscles between our ribs contract to increase the volume of our chest, which causes air to enter our lungs. During the second phase, which is called ‘post-inspiration’, we begin to exhale by relaxing the diaphragm and rib muscles, which causes air to slowly leave the lungs. Post-inspiration is particularly important for generating speech. During the third phase, which is called ‘active expiration’, other muscles contract to actively push air out of the lungs. Active expiration is important during exercise, but is not necessary under resting conditions.

Within the brainstem, neurons working in a network called the preBötzinger complex generate the basis of our breathing rhythm (Gray et al., 2010). These neurons, which are identified as Dbx1 neurons, continue to generate rhythmic nerve impulses, even when they are isolated from the rest of the brain. These nerve impulses normally travel through the brain stem, to cranial nerves, and down the spinal cord (via motor neurons) to the diaphragm and rib muscles. However, this network is primarily active in the inspiration phase (Carroll et al., 2013), so there is considerable interest in efforts to identify and understand the neurons and areas of the brain that are responsible for generating all three phases of the breathing cycle. Now, in *eLife*, Paul Gray and co-workers at the Washington University School of Medicine, Baylor College of Medicine and the College of William and Mary have used elegant genetic approaches to provide new insights into the generation of the breathing rhythm and its different phases.

Gray and colleagues—including Srinivasan Tupal as first author—have built on recent research on mice that identified a second rhythm-generating region in the brainstem. This rhythmogenic region is formed from two overlapping groups of neurons: the retrotrapezoid nucleus (RTN for short) and the parafacial respiratory group (pFRG; Pagliardini et al., 2011). Deleting a gene to block the development of neurons called the Atoh1 neurons in the RTN/pFRG region caused mice to die shortly after birth because they were unable to establish a normal respiratory pattern (Rose et al., 2009). However, this discovery did not identify whether these neurons are directly involved in the generation of active expiration or through another role.

Now, by simultaneously measuring different respiratory motor neurons, Tupal et al. demonstrated that elimination of Atoh1 neurons throughout the ventral respiratory column (a larger region within the brainstem which includes the RTN/pFRG) causes the inspiratory and active expiratory phases of the breathing rhythm to become out of sync with one another (Tupal et al., 2014). However, there was still rhythmic breathing activity. And thus, unlike the loss of the Dbx1 neurons in the preBötzinger complex, losing the Atoh1 neurons in the RTN/pFRG region did not cause the breathing rhythm to be lost.

Tupal et al. conclude that two distinct populations of neurons are responsible for two different functions: some are responsible for generating the different rhythms of breathing, whilst others ensure that these different rhythms remain coordinated. Identifying which genes help to make these neurons, and the roles that these neurons play, may help us to understand medical conditions where breathing rhythms become uncoordinated, such as obstructive sleep apnea (Ramirez et al., 2013). However, the current model still leaves some questions unanswered. For example, how do neurons interacting in the brainstem give rise to the post-inspiration phase of breathing?

The findings by Tupal et al. also offer the thought-provoking possibility that there exists a third rhythmogenic region that generates the rhythm behind post-inspiratory activity. In addition to the preBötzinger complex and RTN/pFRG region, Dbx1 and Atoh1 neurons are also found throughout the ventral respiratory column (Gray, 2013). Thus, one may hypothesize that additional unidentified rhythmogenic networks of neurons may be present in this larger region and that these networks could generate the rhythm of post-inspiration.

If so, and much like what happens for active expiration, these networks would also need to be coordinated with the other networks that control the rhythms of the other phases of breathing. However, the neurons that might coordinate these rhythms are also currently unknown.

Tupal et al. propose that rhythmogenic networks could be organized in segments throughout the ventral respiratory column. This idea has been proposed for the spinal cord (Grillner, 2011) and is supported by experimental evidence (Wiggin et al., 2012). However the suggestion that a similar system in the brainstem exists in the brainstem remains to be validated.

While many questions remain unanswered, the work of Tupal et al. has expanded what we know about the neuronal activity and rhythms that control our breathing—a process that is vital to all of our lives.
